# Predicting the strain-mediated topological phase transition in 3D cubic ThTaN_3_

**DOI:** 10.3762/bjnano.9.132

**Published:** 2018-05-11

**Authors:** Chunmei Zhang, Aijun Du

**Affiliations:** 1 School of Chemistry, Physics and Mechanical Engineering, Queensland University of Technology, Gardens Point Campus, QLD 4001, Brisbane, Australia

**Keywords:** Dirac cone, strain, ThTaN_3_, topological insulator

## Abstract

The cubic ThTaN_3_ compound has long been known as a semiconductor with a band gap of approximately 1 eV, but its electronic properties remain largely unexplored. By using density functional theory, we find that the band gap of ThTaN_3_ is very sensitive to the hydrostatic pressure/strain. A Dirac cone can emerge around the Γ point with an ultrahigh Fermi velocity at a compressive strain of 8%. Interestingly, the effect of spin–orbital coupling (SOC) is significant, leading to a band gap reduction of 0.26 eV in the ThTaN_3_ compound. Moreover, the strong SOC can turn ThTaN_3_ into a topological insulator with a large inverted gap up to 0.25 eV, which can be primarily attributed to the inversion between the d-orbital of the heavy element Ta and the p-orbital of N. Our results highlight a new 3D topological insulator with strain-mediated topological transition for potential applications in future spintronics.

## Introduction

The ThTaN_3_ compound generally presents three structural phases in cubic perovskite (c-PV), hypothetic orthorhombic perovskite (o-PV GdFeO_3_-type), and post-perovskite (PPV) forms [1]. Among them, c-PV ThTaN_3_ was first synthesized in early 1995 [2] and is known to crystallize in the space group *Pm*3*m* with a band gap of approximately 1 eV [1]. Pressure can induce a phase transition from c-PV to o-PV and PPV accompanied by the transition from a moderate band gap semiconductor (≈1 eV band gap in c-PV) to a small band gap semiconductor (PPV) in ThTaN_3_ [1]. c-PV ThTaN_3_ has also been proposed as a potential ground for studying nonlinear optical response [2] due to its large band gap and non-centrosymmetry. As protons are found to be significantly stable in nitrides, c-PV ThTaN_3_ is also evaluated as an ideal proton-conducting ceramic [1]. Nevertheless, theoretical understanding of the electronic properties of ThTaN_3_ is so far very limited and mainly focused on pressure-induced phase transition [3]. Therefore, a systematic study of the electron structure of ThTaN_3_ in a certain phase is highly desired.

Topological insulators (TIs) have attracted much attention due to their distinct quantum mechanical properties, which makes them important in the fields of physics [4–5], chemistry, and materials science [6]. TIs are materials with a bulk band gap generated by strong spin–orbit coupling (SOC) that have topologically protected metallic surface states. Although many materials are theoretically predicted to be TIs [7–11], the experimental realization of TIs is very limited. Therefore, the search for experimentally synthesized large band gap TIs is of paramount importance for their practical application. Theoretically, the transition from the trival insulator to the topological insulator can be achieved by increasing the SOC or by altering the lattice parameters [12–13]. A number of compounds [14–25], such as LaPtBi, LuPtSb, YPdBi [15–18], and HgTe [19–20], have been studied using a first-principles approach, showing that they can be turned into TIs under external strain. All these materials possess heavy elements and the strong SOC can induce a band inversion, which is a typical mechanism for TIs [26–27].

The experimentally observed pressure-induced phase transition in ThTaN_3_ indicates that the electronic structure of 3D ThTaN_3_ is likely very sensitive to the external strain. In particular, c-PV ThTaN_3_ can crystallize in the tetragonal shape with C_4_ rotational symmetry, which is an ideal platform to study its topological properties [28]. The combination of such C_4_ rotational and time-reversal symmetry and the heavy elements (Th, Ta) in ThTaN_3_ are expected to substantially alter the electronic band structure and thus achieve an exotic topological property [26].

By using first-principles calculations, we demonstrate here, for the first time, that the cubic perovskite ThTaN_3_, a relatively large band gap semiconductor, can turn into a TI under moderate pressure/strain. A Dirac cone can emerge in the ThTaN_3_ compound with an ultrahigh Fermi velocity under an 8% compressive strain. The band gap opening, induced by SOC, can be as high as 0.25 eV, which is large enough for the realization of the quantum spin Hall (QSH) states at room temperature. In addition, by tuning the SOC strength, we predict that the topological feature actually starts to show up at a 5% compressive strain. The strain-mediated topological phase transition in the perovskite ThTaN_3_ compound is attributed to band inversion between the d-orbital of the heavy elements and the p-orbital of the N atom [12,29–30].

## Computational Methods

First-principles calculations were performed based on density functional theory (DFT) as implemented in the plane wave basis VASP code [31–33]. A generalized gradient approximation (GGA) in the Perdew, Burke, and Ernzerhof (PBE) form exchange–correlation functional was used. The hybrid Heyd–Scuseria–Ernzerhof (HSE06) functional [34–35] was adopted for the accurate calculation of band structures of 3D ThTaN_3_. A plane-wave basis set with an energy cut-off of 500 eV was employed and long range van der Waals dispersion [36] was incorporated to correct the total energy. The geometry structures were fully optimized until the maximum energy and force were less than 10^−6^ eV and 0.01 eV/Å, respectively. A Monkhorst–Pack *k*-point mesh of 7 × 7 × 7 was used for geometry optimization. The SOC effect was also considered in the calculation. The electron effective mass (m*) of ThTaN_3_ at the conduction band minimum (CBM) is estimated from the curvature of the electronic band dispersion, that is, the formula


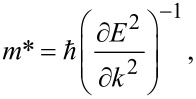


where *E* and *k* are the band energy and reciprocal lattice vector. For anisotropic materials, 
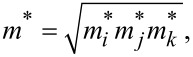
 where *i*, *j* and *k* label the transport direction along the *x*, *y* and *z*-axis.

## Results and Discussion

**Figure 1:**
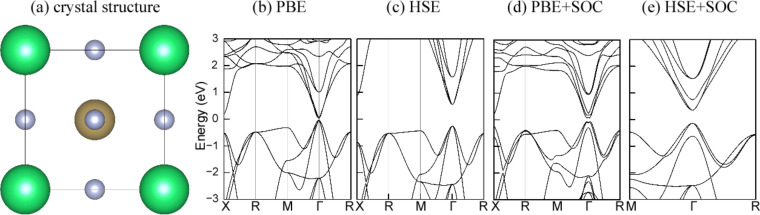
(a) Top view of ThTaN_3_ with green, grey, and brown spheres representing Th, N, and Ta atoms, respectively. (b–e) The band structures of 3D cubic ThTaN_3_ calculated by PBE, HSE, PBE+SOC, and HSE+SOC methods, respectively.

The geometry structure of cubic perovskite ThTaN_3_ was first fully relaxed as shown in Figure 1a. It crystallizes in the space group *Pm*3*m* with C_4_ rotational symmetry. The lattice parameters of c-PV ThTaN_3_ were then calculated by using the PBE functional and the hybrid HSE06 functional methods, respectively. It was found that the PBE functional overestimates the experimental lattice constants by 1%, whereas the HSE06 can successfully reproduce the experimentally reported lattice parameters (4.02 Å) [2].

Figure 1 presents the detailed electronic band structure of 3D ThTaN_3_ for the PBE exchange correlation (Figure 1b) and HSE hybrid functional (Figure 1c). A small direct band gap of 0.07 eV at the Γ point is predicted by the PBE functional and the HSE functional produced a large band gap of 0.76 eV, which is very close to the experimental measurement (1.0 eV) [1]. The band gap should exhibit substantial differences depending on the relative weights of the Hartree–Fock and traditional LDA or GGA exchange energies in the hybrid functional as well as those of the long range van der Waals interactions. However, we found that the impact of van der Waals interaction on the band gap of ThTaN_3_ is negligible. Figure 1d and Figure 1e present band structures in the presence of the effect of SOC. Clearly, an energy gap of 0.15 eV and 0.49 eV were opened by the SOC for the PBE and the HSE functional methods, respectively. Compared to the HSE result without SOC (Figure 1c), the band gap reduction is significantly high (0.26 eV) after the incorporation of SOC.

**Figure 2:**
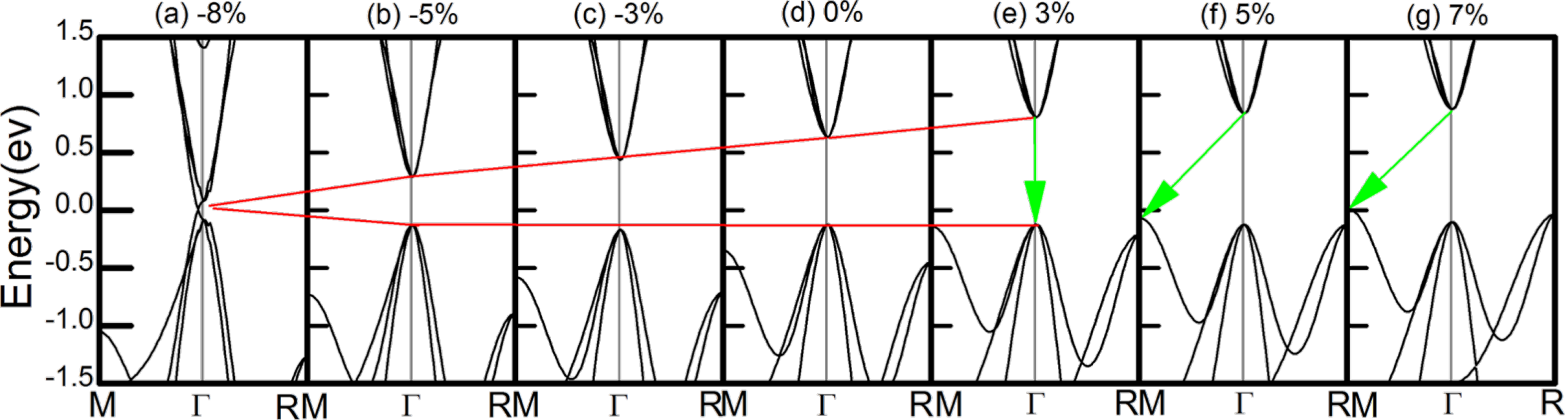
The modulation of band gap (red line) by hydrostatic and tensile strain (−8% to +7%) in ThTaN_3_ by using the HSE method. A Dirac cone emerges when a −8% strain is added to the ThTaN_3_ compound. And the green arrow shows that the change from a direct band gap to an indirect band gap with strain is increased from 3% to 7%. The Fermi level is set at an energy of zero.

Then we turned to study the effect of strain [37] on the electronic structure of c-PV ThTaN_3_ by applying a hydrostatic strain ranging from −10% (compressive strain) to +15% (tensile strain) on 3D ThTaN_3_. As shown in Figure 2d–g, the size of the direct gap continued to increase as the positive strain was increased. At a strain of 3%, the direct band gap turned to an indirect one and the band gap slightly decreased with further increasing strain. When a compressive strain was exerted into 3D ThTaN_3_, the band gap could be significantly reduced. As shown in Figure 2a, the energy gap was reduced to 0 eV at a compressive strain of −8%. A Dirac-cone-like band structure [38] emerged with an ultrahigh Fermi velocity 6.33 × 10^5^ m/s that is comparable to that of graphene (1.1 × 10^6^ m/s) [39]. It is very important to note that the conduction band (CB) of ThTaN_3_ is very dispersive around the Γ point, signifying a very low electron effective mass. The effective mass of the electron at the Γ point is calculated to be 0.395 *m*_e_. Such a small electron mass will greatly improve charge carrier mobility, suggesting great potential for application of ThTaN_3_ in electronics.

**Figure 3:**
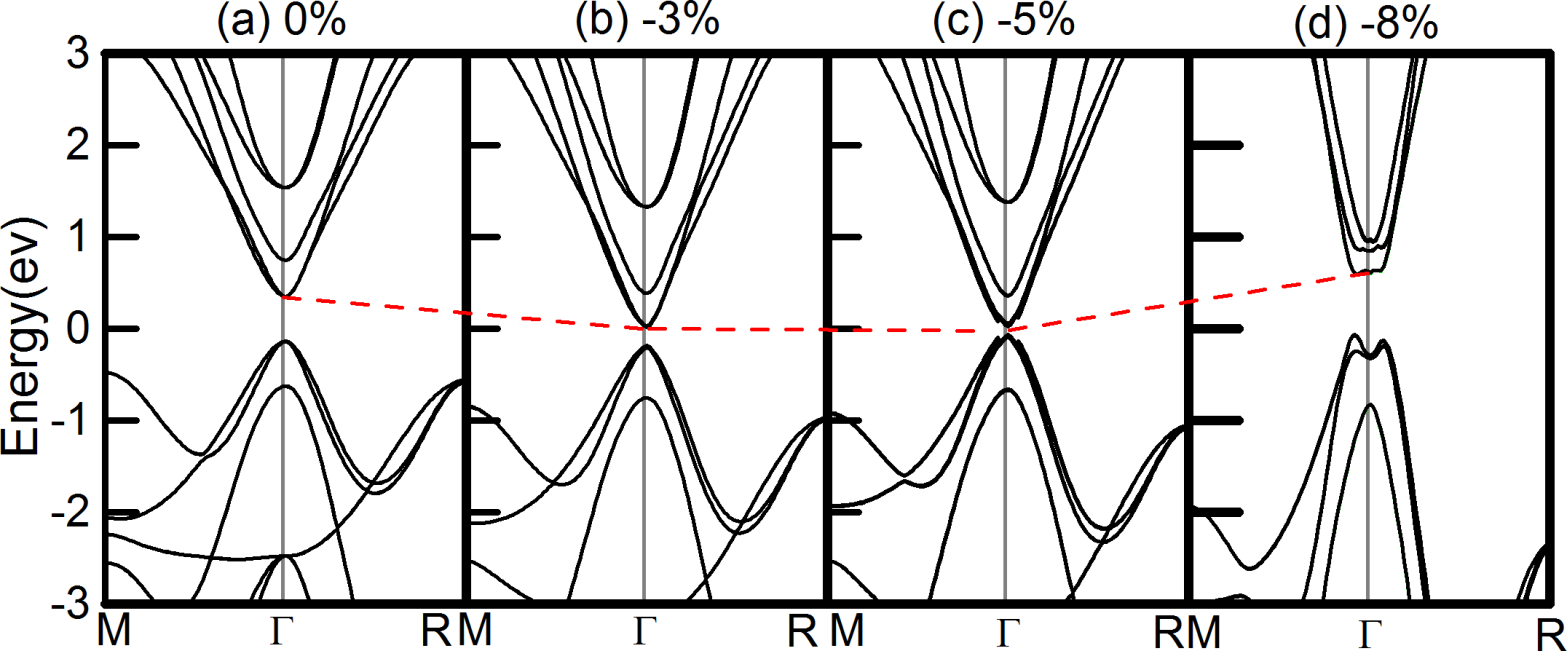
Band structures of ThTaN_3_ calculated by the HSE+SOC method under a strain of −8% to 0%. The Fermi level is set to zero.

As eluded to above, the effect of SOC on the band gap of ThTaN_3_ is significant. It is therefore important to further study the effect of strain on the electronic structure of ThTaN_3_ in the presence of SOC (Figure 3). For strain-free ThTaN_3_, the band gap is 0.49 eV as calculated by the HSE+SOC method. The band gap is reduced approximately 0.26 eV compared to the HSE result (0.76 eV) without SOC. When an 8% compressive strain is exerted on the ThTaN_3_ compound, SOC opens a large band gap (approximately 0.25 eV) for the Dirac cone as shown in Figure 3d. It can be seen that under compressive strain, the SOC gap of ThTaN_3_ can be closed and reopened. In addition, the shape of the band structure is changed correspondingly, indicating a topological phase transition [40–42]. In order to determine topological features, we calculated the Z2 topological index [19,27]. The topological invariant Z_2_ is 1;(0,0,0) for ThTaN_3_ under 8% compressive strain, which indicates the strong topological property (more details including methods and parities of the relevant bands can be found in Supporting Information File 1). We further scrutinized the SOC band structure of ThTaN_3_ (Figure 3c) and find that the band inversion actually occurred at a 5% compressive strain. The above results clearly indicated that we can turn ThTaN_3_ into a TI by applying an external hydrostatic pressure in the presence of SOC.

**Figure 4:**
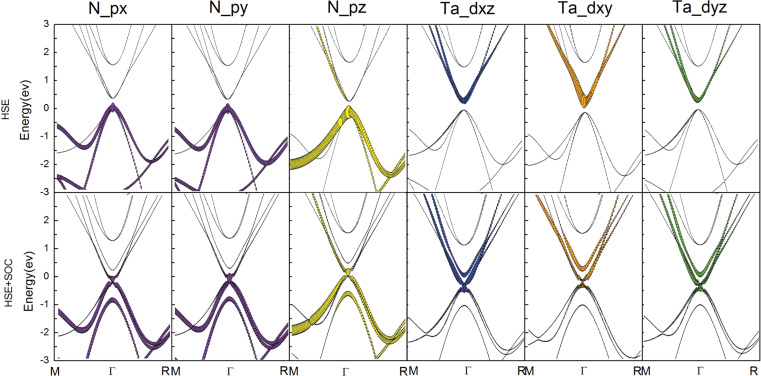
Orbital-resolved band structures for ThTaN_3_ under 5% compressive strain as calculated by the HSE (top panel) and HSE+SOC methods (bottom panel). The contributions are mainly from p_x_, p_y_, p_z_ of N atoms and d_xz_, d_xy_, d_yz_ of Ta atoms. The thicker and thinner lines account for large and low orbital contributions, respectively.

We further analyzed the orbital-resolved band structure of ThTaN_3_ at a lower compressive strain (5%) as shown in Figure 4. The conduction band (CB) state mainly consisted of p_x_, p_y_ and p_z_ orbitals of the N atom, while the valence band (VB) state is comprised of the hybridization d_xy_, d_xz_, d_yz_ orbitals of the Ta atom. When the SOC effect was incorporated, a p–d-type band inversion took place with the reduction of the band gap, manifesting a topological phase transition due to the synergistic effects of SOC and lattice strain.

**Figure 5:**
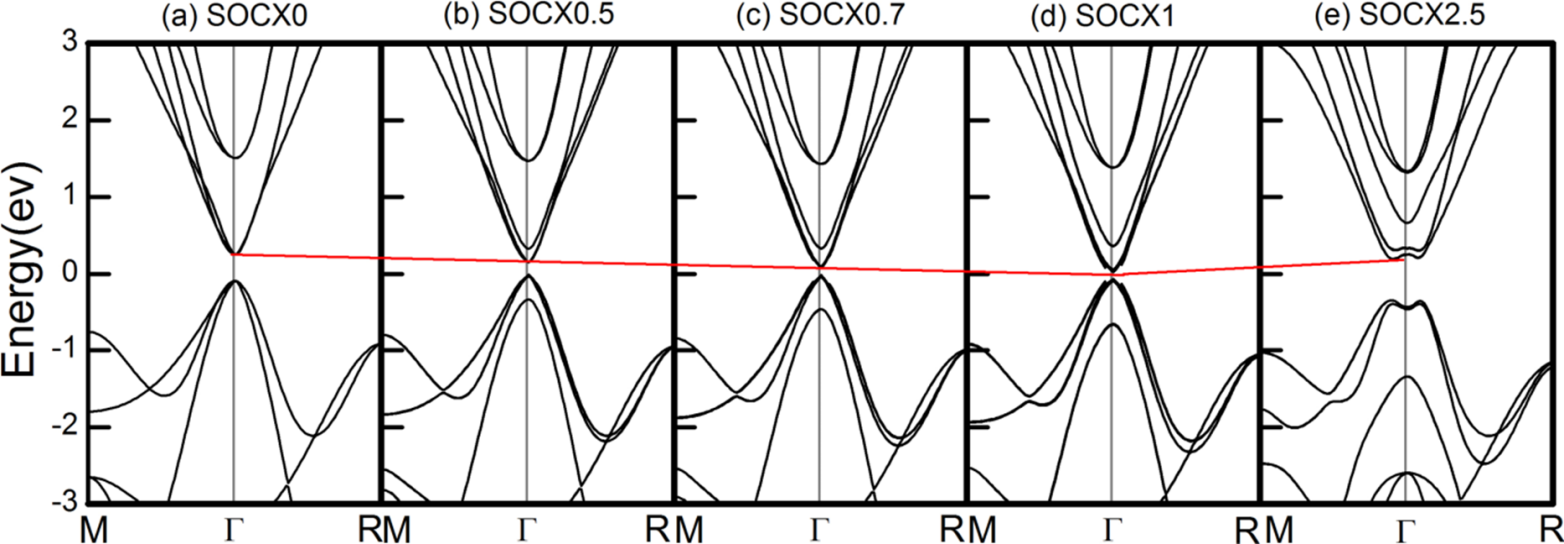
Band structures for 3D cubic ThTaN_3_ with a 5% compressive strain as calculated by the HSE+SOC method at different SOC strengths (0–2.5 times). The Fermi energy level is set to zero.

Generally, the topological phase transition can be also directly observed by modifying the SOC strength [29–30]. To provide a clear picture of the band inversion or topological phase transition in ThTaN_3_ under a 5% compressive strain, we then studied the electronic structure of 3D cubic perovskite ThTaN_3_ at various SOC strengths as shown in Figure 5a–e. With increasing SOC, the VBM and CBM gradually become closer (see Figure 5a–e), and then the gap closed and reopened with a large portion of band inversion, which can be clearly seen from the enormous change of the CBM and VBM band shape into Mexican-hat-like band dispersion, a typical indication of topological phase transition (see Figure 5e).

## Conclusion

In summary, we have systematically studied the electronic structure in the 3D perovskite ThTaN_3_ compound. We find the band gap of ThTaN_3_ is very sensitive to the lattice strain. A Dirac-cone-like band with an ultrahigh Fermi velocity can emerge at a compressive strain of 8%. The topological phase transition can be realized with a large gap (≈0.25 eV) opened in the presence of SOC. Further analysis of orbital contribution indicates p–d band inversion in 3D ThTaN_3_. Our results highlight a new, interesting, 3D, topological insulator material with great potential for future application in spintronics.

## Supporting Information

File 1 Additional calculations.
The lattice parameters of ThTaN_3_ under strain, the surface state of ThTaN_3_, and the calculation of the topological invariant number Z2.
