# Supplementary material for: Predicting the strain-mediated topological phase transition in 3D cubic ThTaN3
Source: Beilstein J Nanotechnol. 2018 May 11;9:1399–404. doi: 10.3762/bjnano.9.132 (PMC6009352; doi:10.3762/bjnano.9.132)
Supplement: File 1 — Additional calculations. The lattice parameters of ThTaN3 under strain, the surface state of ThTaN3, and the calculation of the topological invariant number Z2. [file Beilstein_J_Nanotechnol-09-1399-s001.pdf]

Supporting Information

for

Predicting the strain-mediated topological phase transition in 3D cubic $ThTaN_3$

Chunmei Zhang and Aijun Du*

Address: [1]School of Chemistry, Physics and Mechanical Engineering, Queensland University of Technology, Gardens Point Campus, QLD 4001, Brisbane, Australia

*Corresponding author

Email: aijun.du@qut.edu.au - Aijun Du

Additional calculations

Table S1: Lattice parameters of $ThTaN_3$ under strain.

	-8%	-5%	-3%	0	3	5	7
Lattice parameters (Å) (a=b=c)	3.69	3.81	3.89	4.01	4.13	4.21	4.29

Calculation of Z_2

In order to determine topological features, we used the parity criteria [S1] to calculate the Z_2 topological index. The Z_2 index is determined by the parity of occupied bands on each time-reversal invariant momentum. In this strategy, the Z_2 invariant is defined by

$$(-1)^{v} = \prod_i \delta_i = -1$$

$$\delta_i = \prod_{m=1}^{N} \varepsilon_{2m}(\Gamma_i)$$

For 2N occupied bands. $\varepsilon_{2m}(\Gamma_i) = \pm 1$ is the parity eigenvalue of the 2m-th occupied energy bands at the time-reversal invariant momentum Γ_i. With the inversion symmetry, the Z_2 topological invariants can be deduced from the knowledge of the parities of the eight time-reversal and parity invariant points at Brillion (BZ). This provides a simple method for determining the topological phases of the lattices with inversion symmetry, without having to know about the global properties of the energy bands.

For the $ThTaN_3$ compound, the eight time reversal invariant momenta are (0,0,0), (0,5,0,0), (0,0.5,0), (0,0,0.5), (0.5,0.5,0), (0.5,0,0.5), (0,0.5,0.5), (0.5,0.5,0.5) in BZ, as shown in Table S2. The parties of a band at the eight time-reversal momenta is determined from the corresponding electron wavefunctions given by first-principle calculations using the Quantum ESPRESSO package [S2].

Table S2: The valence band parity at the eight time reversal invariant momenta for $ThTaN_3$ under 8% compressive strain.

valence band parity	Γ(0,0,0)	X(0.5,0,0) =(0, 0.5, 0) =(0, 0, 0.5)	M(0.5,0.5,0) =(0.5,0,0.5) =(0,0.5,0.5)	R(0.5,0.5,0.5)
1	+	-	+	+
2	+	-	-	+
3	+	+	-	+
4	-	-	+	-
5	-	+	+	-
6	-	+	+	-
7	-	+	+	-
8	-	-	+	-
9	-	-	-	-
10	+	+	-	-
11	+	+	-	-
12	+	+	+	-
δ_i	+1	$(-1)^3$	$(-1)^3$	-1

For this system, the four independent Z_2 invariants [S3] are 1;(0,0,0) with $v_0 = 1$, thus, $ThTaN_3$ is a strong topological insulator under 8% compressive strain.

Surface state

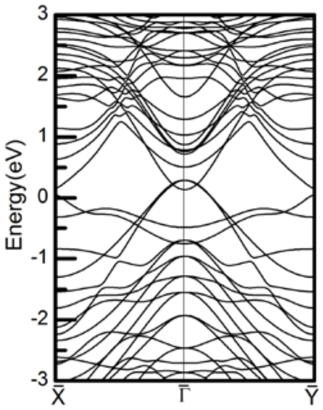

Figure S1: The (001) surface state of $ThTaN_3$, which can be clearly seen around Γ point. The high-symmetry points $\overline{X}$, $\overline{\Gamma}$, and $\overline{Y}$ of the surface Brillouin zone are labelled.

It should be noted that there are supposed to have two Dirac cones at the Fermi surface, but the shape as shown in Figure S1 is somewhat parabolic. This is mainly attributed to that a five-layer (~20Å) surface model (too thin) is adopted for the surface state calculation due to the limitation of our current computational capability.

References

S1. Fu, L.; Kane, C. L. *Phys. Rev. B* **2007,** 76, 045302.

S2. Giannozzi, P. *J. Phys.: Condens. Matter* **2009,** 21, 395502.

S3. Moore, J. E.; Balents, L. *Phys. Rev. B* **2007,** 75, 121306.
